# Retinoic Acid-Induced Transglutaminase 2 Expression Reduces Sensitivity to Cisplatin in the Hormone-Positive MCF-7 Breast Cancer Cell Model

**DOI:** 10.3390/ijms26168101

**Published:** 2025-08-21

**Authors:** Ebidor U. Lawani-Luwaji, Claire V. S. Pike, Peter J. Coussons

**Affiliations:** 1 Department of Medical Laboratory Sciences, College of Health Sciences, Niger Delta University, Wilberforce Island, PMB 071, Amassoma 560103, Bayelsa State, Nigeria; ebilawani-luwaji@ndu.edu.ng; 2 School of Life Sciences, Faculty of Science and Engineering, Anglia Ruskin University, Cambridge CB1 1PT, UK; claire.pike@aru.ac.uk

**Keywords:** breast cancer, cisplatin, dietary retinoids, transglutaminase 2, MCF-7

## Abstract

Cisplatin is an effective chemotherapeutic drug, but is limited both by its toxicity and its tendency to induce drug resistance rapidly in some patients. Tissue transglutaminase 2 (TG2), which is overexpressed in various cancers, has two main isoforms: a long (TG2-L) and a short form (TG2-S). While TG2-L supports cell survival, conversely, TG2-S promotes cell death. Evidence increasingly suggests that TG2 may be a suitable target for combating chemoresistance in a variety of human cancers. Here, we show that cisplatin toxicity towards wild-type MCF-7 breast cancer cells is associated with reduced TG2-L and TG2-S expression, whereas approximately doubling the TG2-L expression through the retinoic acid pre-treatment of these cells induces survival in the presence of cisplatin at levels similar to those seen in long-term cisplatin-co-cultured cells, which have reduced sensitivity. The treatment of cisplatin-surviving cells with cisplatin alone did not significantly alter the levels of either TG2 isoform, whereas the cisplatin challenge of cisplatin-surviving MCF-7 cells following 20 µM retinoic acid pre-treatment resulted in increased levels of TG2-L, increased TG2 enzyme activity, and no significant change in TG2-S levels, with increased cell survival. These findings suggest a subtype-specific regulatory effect of RA in cisplatin-surviving MCF-7 cells, with TG2-L upregulated at higher RA concentrations, potentially contributing to altered cisplatin sensitivity. Anti-TG2 siRNA silencing reduced cisplatin IC50 to base levels in both wild-type and cisplatin-surviving MCF-7 cells, supporting the notion that the modulation of TG2 expression could offer a significant benefit to cisplatin efficacy. Preventing excessive retinoic acid exposure may also be a mechanism for maximising cisplatin efficacy, considering TG2 modulation.

## 1. Introduction

Retinoic acid (RA), the biologically active metabolite of vitamin A, is important in regulating cell growth and differentiation. While vitamin A is essential for physiological functions such as vision, immunity, and reproduction, its metabolite RA exerts its effects through nuclear retinoic acid receptors (RARs) and retinoid X receptors (RXRs). These heterodimers regulate transcription by binding to retinoic acid response elements (RAREs) in target genes [1,2]. Vitamin A, predominantly stored as retinyl esters in the liver and other tissues, is metabolised into RA through a tightly regulated process. All-trans retinoic acid (atRA) is the primary active metabolite of retinol and is responsible for most physiological functions. Evidence suggests that elevated levels of RA may influence cancer biology by modulating gene expression.

One such target is tissue transglutaminase 2 (TG2), a multifunctional enzyme involved in multiple cellular processes, including differentiation, apoptosis, and tissue repair [3]. TG2 catalyses the post-translational modification of amide and amine moieties of glutaminyl- and lysyl-side chains in the presence of intracellular Ca^2+^ [4]. In some cases, TG2 can introduce isopeptide bonds between polypeptide chains. Such bonds have great physiological significance because of their stable nature and resilience to mechanical and proteolytic breakdown [5]. TG2 contributes to the modulation of the cell cycle, cellular adhesion, endocytosis, and the formation of apoptotic bodies during programmed cell death. The enzyme is overexpressed in cancer and chemoresistant cells and is strongly implicated in mechanisms that induce chemotherapeutic resistance, correlating with poor therapeutic outcomes [6,7,8].

TG2 exists in two isoforms, generated by alternative splicing, with opposing roles: TG2-L, which supports cell survival and contributes to tumour progression and chemoresistance, and TG2-S, which promotes cell death and may act as a tumour suppressor [9,10]. The primary contrast between these isoforms is found in the sequence of amino acids at the C-terminus. Specifically, the long variant possesses a GTP-binding domain, which enables it to be classified as a small G-protein. On the other hand, the short variant lacks this domain and is believed to be consistently active [7].

Studies show that RA increases the expression of transglutaminase 2 (TG2), as seen in different cell types: neuroblastoma cells [11], glial cells [12], macrophages [13], rat hepatocytes [14], and human hepatocarcinoma cells, where it has been implicated in contributing to chemoresistance during chemotherapy [15]. While RA is vital for normal cellular function, its elevated levels, often influenced by dietary vitamin A intake, might increase TG2 expression, complicating treatment effectiveness.

Cisplatin is a key chemotherapeutic agent used in the treatment of various cancers, including hormone-positive breast cancer. Despite its efficacy, cisplatin is plagued by significant drawbacks, including dose-limiting toxicities such as gastro-toxicity, nephrotoxicity, ototoxicity, and myelosuppression, as well as hypersensitivity reactions [16,17] and cancer cells developing resistance to cisplatin over time. Researchers have explored combination therapies to address these challenges, which have shown promise in overcoming resistance [16]. Cisplatin has been used with other platinum-based drugs to treat human cancers, such as oesophageal [18], ovarian [19,20], cervical [21,22], bladder, blood, sarcoma, lung, testicular, and breast cancers [20,23]. While in the context of breast cancer, it is classically used to treat cases of triple-negative disease, emerging evidence supports the experimental and clinical relevance of cisplatin in ER⁺ breast cancer models, particularly in tamoxifen-resistant or BRCA1-mutant contexts [24]. For example, the transcriptomic profiling of cisplatin-resistant MCF-7 cells demonstrates key gene expression changes associated with reduced cisplatin sensitivity in ER⁺ backgrounds [25]. Cisplatin has also been shown to reduce the survival of ER⁺ breast cancer cells while promoting the differentiation of progenitor subsets [25], and clinical trials such as NCT02221999 [26] have included ER⁺/PR⁺ breast cancer patients in neoadjuvant cisplatin arms, suggesting that cisplatin’s utility may extend beyond triple-negative disease. Additionally, innovative approaches encapsulating cisplatin in nanoparticles or using platinum (IV) prodrugs designed to minimise systemic toxicity and enhance targeted delivery to tumours have been explored to reduce systemic toxicity and improve tumour-targeted delivery [27,28].

In this study, we investigate the relationship between RA-induced TG2 expression in an MCF-7 breast cancer cell line and sensitivity to cisplatin, observing that while increased TG2-L expression is strongly associated with the increased survival of cisplatin treatment, the silencing of TG2 increases cisplatin sensitivity significantly. These findings highlight the importance of retinoid regulation as a potential strategy to help overcome chemoresistance and enhance therapeutic outcomes in breast cancer treatment.

## 2. Results

### 2.1. Establishment of the Inhibitory Concentration of Cisplatin

**Figure 1:**
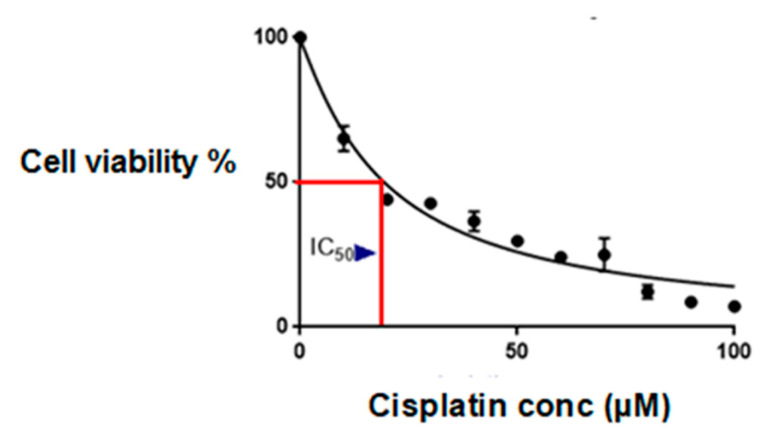
Establishment of the IC_50_ of cisplatin in wild-type MCF-7 cells. The IC_50_ of wild-type MCF-7 cells with the CCK-8 assay was 18 µM, as indicated by the red lines. The data represent the mean ± SEM from three independent experiments.

Figure 1 shows the inhibitory concentration (IC_50_) of cisplatin in wild-type MCF-7 cells with the CCK-8 assay. The IC_50_ was 18 µM, as shown in Figure 1.

#### 2.1.1. Establishment of Cisplatin-Surviving MCF-7 Cells (cMCF-7)

**Figure 2:**
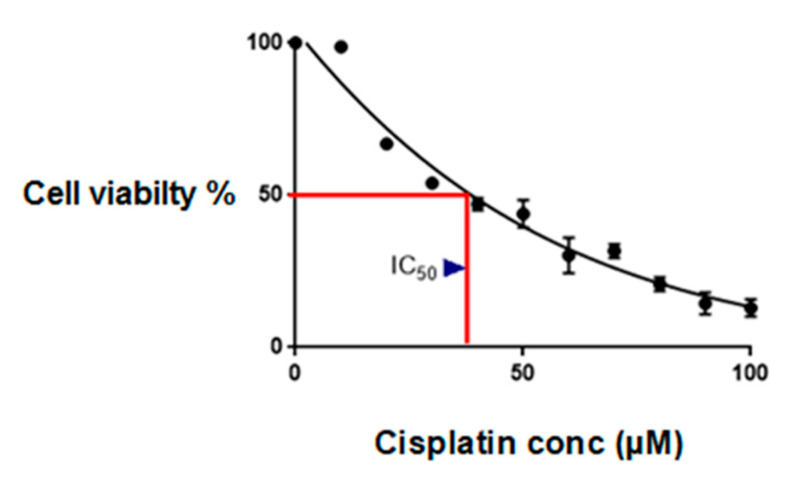
Establishment of the IC_50_ of cisplatin in cisplatin-surviving MCF-7 cells (cMCF-7). The IC_50_ of cisplatin-surviving cells with the CCK-8 assay was 35 µM, as indicated by the red lines. The data represent the mean ± SEM from three independent experiments.

Cisplatin-surviving cells were developed to identify the role played by TG2 isoforms in chemoresistance, as described in the Section 4. The IC_50_ of generated cisplatin-surviving cells (cMCF-7) was 35 µM, as shown in Figure 2. The data represent the mean ± SEM from three independent experiments.

#### 2.1.2. TG2 Levels Are Increased in Cisplatin-Surviving Cells

**Figure 3:**
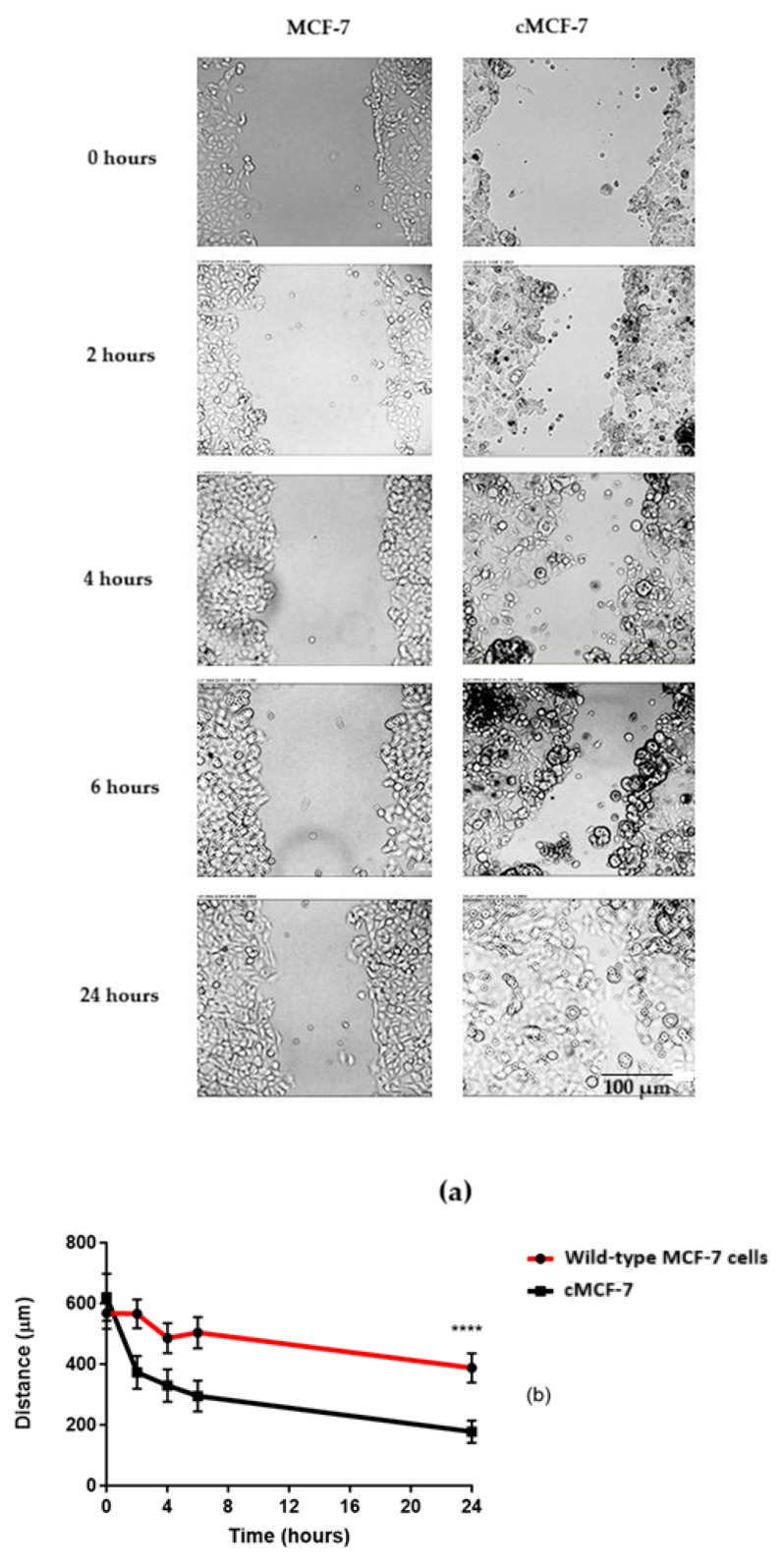
A scratch assay on wild-type (MCF-7) and cisplatin-surviving (cMCF-7) cells. (**a**) Representative images taken with the ZOETM Fluorescent Cell Imager, Scale bar = 100 µm, illustrating both cell types’ healing process over time. (**b**) A line graph showing the distance (in micrometres) covered by the wild-type and cisplatin-surviving cells. Data are expressed as the mean ± standard error of the mean (SEM) from three independent experiments. Statistical analysis revealed a significant difference between the wild-type and cMCF-7 cells, indicated by (****), which denotes a *p*-value of less than 0.0001 as determined by a two-way ANOVA.

A wound-healing assay demonstrated that cisplatin-surviving cMCF-7 cells exhibit a significantly faster migration rate and cover more of the scratch area within 24 h compared to wild-type MCF-7 cells. This assay was performed to further characterise the phenotype exhibiting reduced cisplatin sensitivity, confirming that the cells which were developed in-house not only exhibited reduced sensitivity but also displayed enhanced migratory capacity. (See Figure 3a,b).

**Figure 4:**
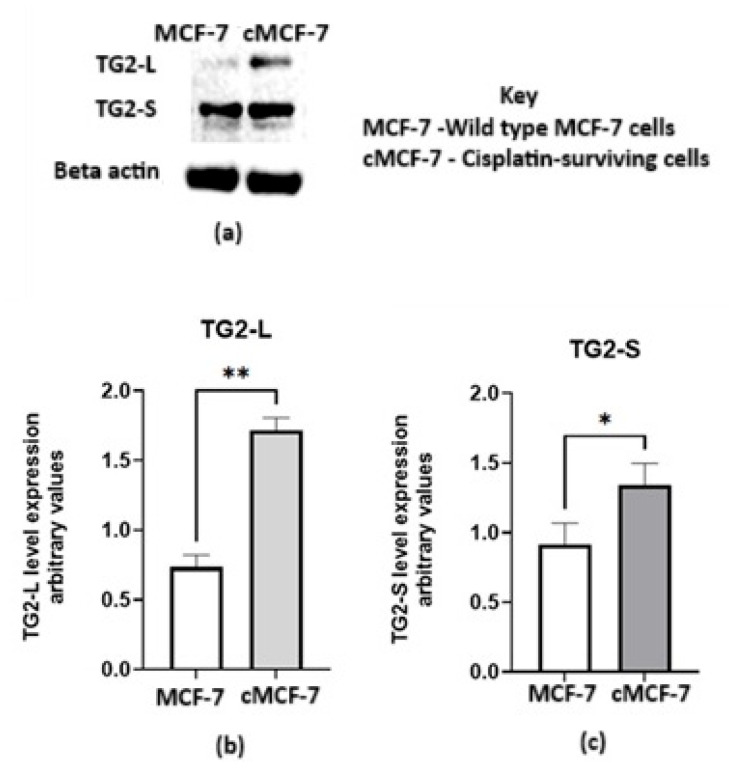
The TG2 protein expression in wild-type (MCF-7) and cisplatin-surviving (cMCF-7) cells. (**a**) A representative Western blot; the histograms represent band quantification for (**b**) the long-form and (**c**) the short-form of the protein. The results are presented as the mean ± SEM from three independent experiments, and significance is denoted by *, with a *p*-value of 0.0286 and ** 0.0016 when analysed with Student’s *t*-test.

To investigate TG2 levels in the cMCF-7 cells, TG2 protein expression in both cisplatin-sensitive and cisplatin-surviving cells was analysed using Western blotting. The findings presented in Figure 4 indicate that TG2-L and TG2-S expression are significantly elevated in cisplatin-surviving cMCF-7 cells compared to wild-type MCF-7 cells.

### 2.2. Retinoic Acid Increases TG2-L Expression

**Figure 5:**
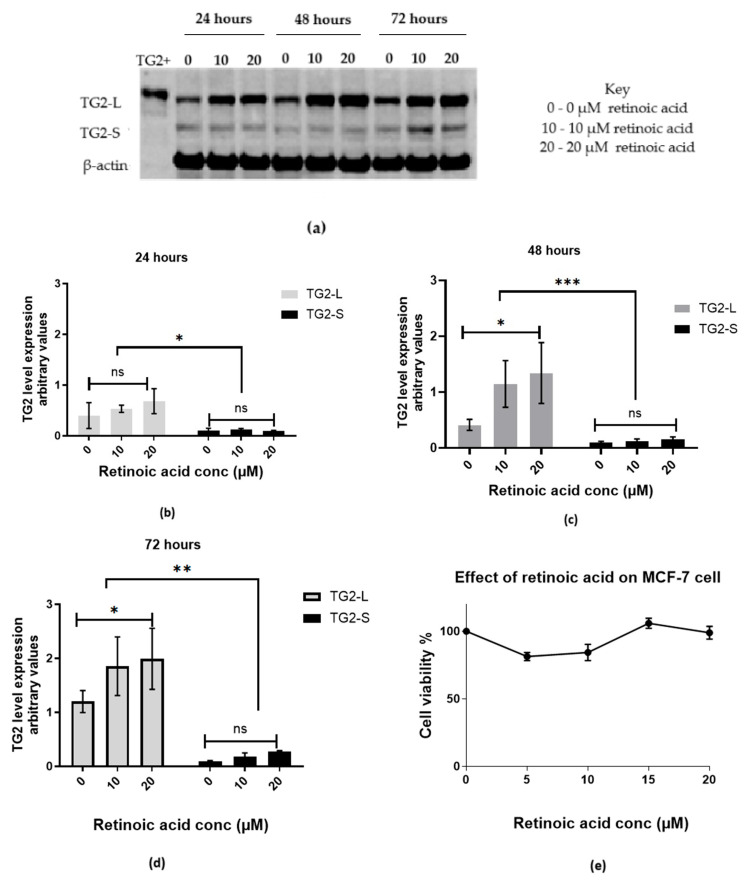
The pattern of TG2 expression in wild-type MCF-7 cells over 72 h of incubation with retinoic acid. (**a**) A representative Western blot showing the induction of TG2 after RA treatment over 72 h at 0, 10, and 20 µM concentrations. The histograms quantify the bands after (**b**) 24 h, (**c**) 48 h, and (**d**) 72 h. β-actin was used as a loading control. The difference in the levels of TG2-L to TG2-S was statistically significant when analysed with the 2-way ANOVA. Significance is denoted by ns (non-significant), * (0.0283), ** (0.0049), and *** (0.0003). The results show no statistically significant differences in TG2-S expression among the different conditions and hours. (**e**) The cell viability of MCF-7 cells treated with RA for 72 h was determined with the MTT assay. The results are presented as the mean ± SEM computed from three independent experiments.

We examined the effect of RA on TG2 isoform expressions over time. The Western blot results (Figure 5a–d) show low TG2-L expression at 24 h across all conditions, approximately doubling by 48 h, particularly at 20 µM RA, while TG2-S remains relatively low. By 72 h, TG2-L expression is at its highest with 20 µM RA, while TG2-S expression increases only slightly, to the extent that it is not statistically significant. These results indicate that RA selectively enhances TG2-L expression over time and in a dose-dependent manner. Cell viability analysis showed that RA is non-toxic to cells within the 0–20 µM concentration range (Figure 5e), ensuring cell viability throughout the experiment.

#### 2.2.1. Retinoic Acid Increases TG2-L Levels and Reduces Cisplatin Cytotoxicity in MCF-7 Wild-Type Cells

**Figure 6:**
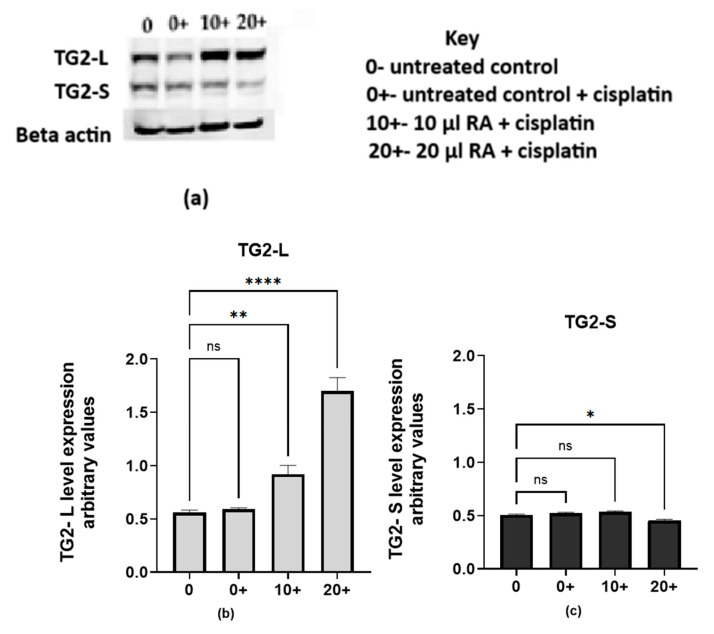

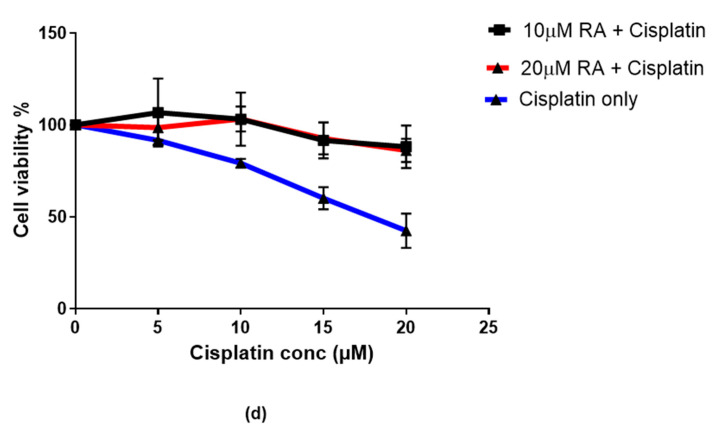
Retinoic acid induces TG2-L expression and reduces cisplatin cytotoxicity in MCF-7 wild-type cells. (**a**) A representative Western blot of wild-type MCF-7 cells treated with RA and cisplatin. β-actin was used as the loading control. TG2-L and TG2-S expression levels were assessed in cells treated with 18 µM cisplatin alone (0+) or combined with untreated cells (0), which served as baseline control. Data are presented as the mean ± SEM (*n* = 3). (**b**) The quantification of TG2-L levels was normalised to β-actin. One-way ANOVA revealed a significant difference among the treatment groups (F(3,8) = 142.2, *p* < 0.0001, R^2^ = 0.9816), with equal variances confirmed by the Brown–Forsythe test (*p* = 0.4330). Dunnett’s post hoc test showed that TG2-L expression significantly increased at 10+ (*p* = 0.0011) and 20+ µM RA (*p* < 0.0001) compared to the untreated control (0). No significant difference was observed between 0 and 0+ (*p* = 0.9271). (**c**) For TG2-S, one-way ANOVA indicated a significant difference among the groups (F(3,8) = 13.16, *p* = 0.0018, R^2^ = 0.8315), with no significant differences in variance (*p* = 0.9910). Dunnett’s test revealed that only the 20+ group showed a significant reduction in TG2-S levels compared to the control (*p* = 0.0133), while 0+ and 10+ were not significantly different (*p* > 0.05). (**d**) The cytotoxicity of cisplatin on the viability of wild-type MCF-7 cells after RA pre-treatment for 72 h was determined by the MTT assay. The results are presented as the means ± SEM from three independent experiments. **Note:** *p* < 0.05 (*), *p* < 0.01 (**), *p* < 0.0001 (****), ns = not significant.

We set out to investigate how elevated levels of TG2-L affect the cytotoxicity of cisplatin. The Western blot results (Figure 6a) and the corresponding band quantification (Figure 6b,c) illustrate the expression levels of both TG2-L and TG2-S under various treatment conditions. In the untreated control (0), TG2-L and TG2-S are at baseline levels. Cisplatin treatment alone (0+) did not significantly change the baseline levels of either isoform. However, when RA was added alongside cisplatin (10+ and 20+), TG2-L levels increased compared to the cisplatin-only group (0+), with the highest expression observed at 20 µM RA. In contrast, when challenged with cisplatin, TG2-S levels were significantly lower in cells pre-treated with 20 µM RA. This shift in the TG2-L to TG2-S ratio suggests that RA promotes TG2-L expression while suppressing TG2-S in cisplatin-challenged cells, potentially contributing to reduced cisplatin sensitivity.

To assess the effect of increased TG2-L levels in RA-pre-treated cells, we determined the inhibitory concentration of cisplatin in this context (see Figure 6d). The data show that cisplatin had a smaller effect on RA-pre-treated cells (indicated by the black and red curves) compared to the untreated controls (blue curve). This reduced sensitivity is likely a result of the increased expression of TG2-L in RA-pre-treated cells.

#### 2.2.2. Retinoic Acid Increases TG2-L Levels and Reduces Cisplatin Cytotoxicity in Cisplatin-Surviving Cells (cMCF-7)

**Figure 7:**
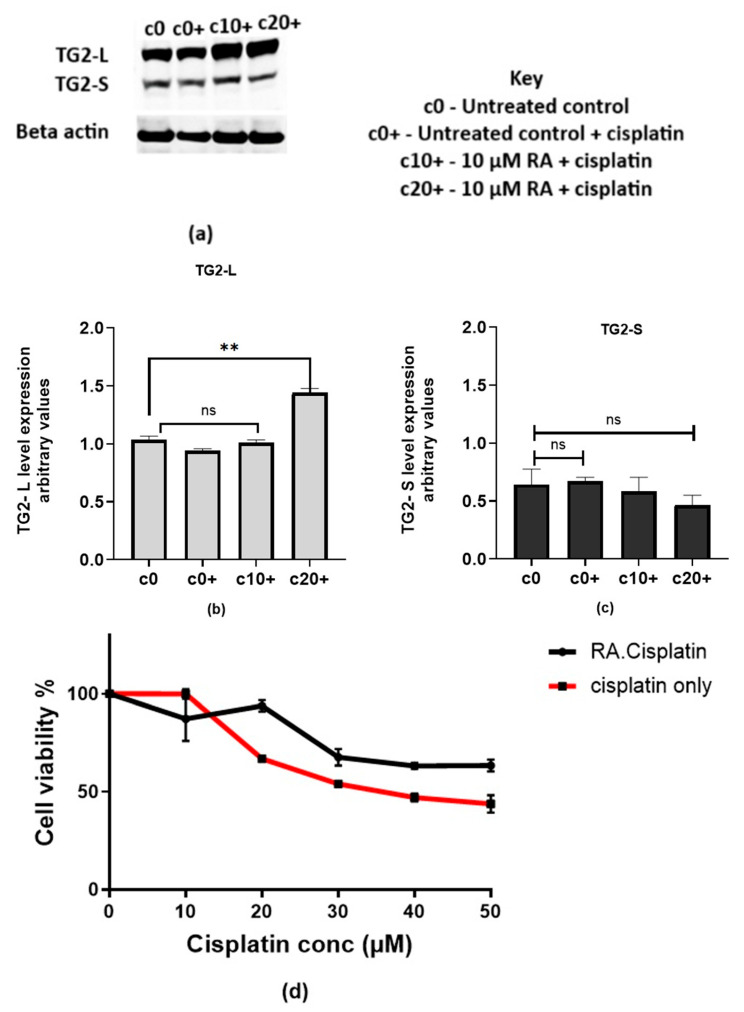
Retinoic acid increases TG2-L levels and reduces the cytotoxicity of cisplatin in cMCF-7 cells. The expression levels of TG2-L and TG2-S were measured in cisplatin-surviving MCF-7 cells treated with 18 µM cisplatin alone (c0+) or in combination with increasing concentrations of retinoic acid (c10+ and c20). Untreated cells (c0) served as a control. (**a**) Representative Western blot. (**b**) One-way ANOVA followed by Dunnett’s post hoc test showed that TG2-L levels were reduced non-significantly with cisplatin alone and increased non-significantly at c10+ but were significantly increased by a high-dose RA pre-treatment (20 µM, *p* = 0.0024). (**c**) In contrast, TG2-S expression exhibited a non-significant downward trend across treatment groups (ANOVA, *p* = 0.1269), with no significant differences detected in pairwise comparisons. (**d**) The cytotoxicity of cisplatin in MCF-7 cells with reduced cisplatin sensitivity, after 72 h of pre-treatment with 20 µM RA, was determined by the MTT assay. RA-treated cells exhibited a higher viability compared to the cisplatin-only group (previous IC_50_ of 35 µM) with *p* < 0.05 and two-way ANOVA with Bonferroni’s post hoc test. Data are presented as the mean ± SEM from three independent experiments. **Note:** *p* < 0.01 (**), ns = not significant.

To examine the effect of increased TG2 levels on cisplatin-surviving MCF-7 cells, they were also pre-treated with RA for 72 h before exposure to cisplatin. The Western blot (Figure 7a) and blot quantification (Figure 7b,c) show that TG2-L levels significantly increased with the cisplatin challenge following RA pre-treatment at a higher concentration (c20+). Conversely, TG2-S expression was not significantly affected by the cisplatin challenge, even when using RA pre-treatment, although higher RA concentrations (c20+) produced a non-significant decline in TG2-S levels.

This pattern suggests that increased TG2-L expression may contribute to further reduced cisplatin sensitivity in these cells (Figure 7d), potentially reducing the effectiveness of the drug. Cell viability decreased with increasing cisplatin concentrations in both treatment groups. However, cells pre-treated with retinoic acid (RA) (black curve) showed modest but consistently higher survival rates compared to the cisplatin-only group (red curve), indicating reduced sensitivity to cisplatin.

### 2.3. TG2 Enzyme Activity

**Figure 8:**
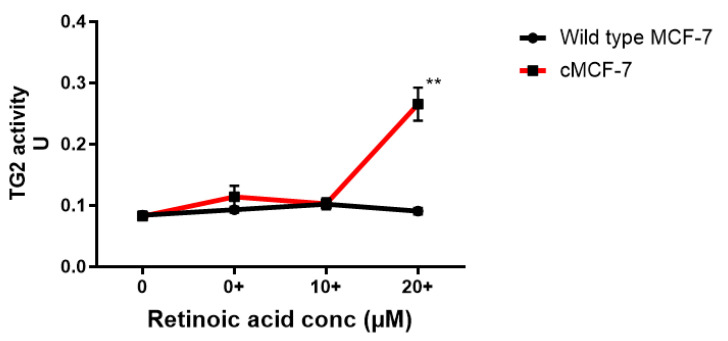
Enzyme activity in retinoic acid pre-treated wild-type and cisplatin-surviving MCF-7 cells after cisplatin treatment. The TG2 activity of wild-type and cisplatin-surviving MCF-7 cells, pre-treated or not, with RA was determined using the TG2 COV assay kit. Statistical significance is denoted by ** (0.0048). The results are presented as the means ± SEM from three independent experiments.

There was no significant difference in TG2 activity between the RA-pre-treated wild-type MCF-7 cells and those without RA pre-treatment (Figure 8). Almost the same pattern is mirrored in the cisplatin-surviving cells, except at the 20 µM RA pre-treatment concentration, where the enzyme activity doubled compared to that of the wild-type cells and the cisplatin-surviving cells without RA pre-treatment—a condition in which the significant up-regulation of TG2-L was also observed (Figure 7b).

### 2.4. Transfection with TG2-Specific siRNA Increases Cisplatin Cytotoxicity in Wild-Type MCF-7 Cells

The results from both the viability and flow cytometry assays indicate that the IC_50_ of cisplatin was lower in cells transfected with siRNA against TG2 compared to non-transfected cells. Cells were treated with lipofectamine to check for toxicity and cell viability before transfection (Supplementary Figure S1); flow cytometry analysis (Supplementary Figure S2) showed that the cells remained healthy.

**Figure 9:**
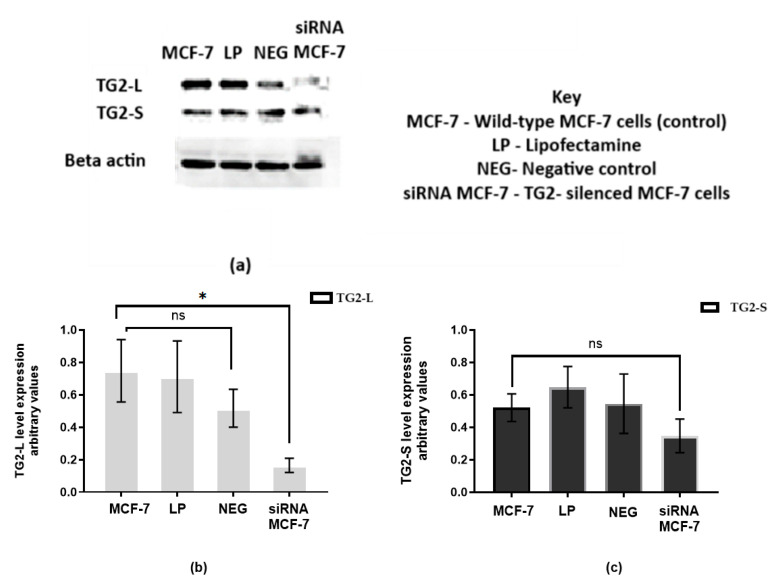
The effect of silencing on TG2 levels in wild-type MCF-7 cells. (**a**) A representative Western blot of TG2 levels in wild-type MCF-7 cells after transfection. β-actin was used as the loading control. The histograms represent the band quantification of (**b**) the TG2-L expression and (**c**) TG2-S expression. Cells treated with siRNA showed a significant reduction in TG2-L expression compared to untreated MCF controls (*p* = 0.0417) with Dunnett’s post hoc test. A reduction was seen in TG2-S, but this was not statistically significant. Normalisation was achieved with β-actin, and the results were computed from the mean of three independent experiments (±SEM). Significance is denoted by * (0.0081), and ns (non-significant) when analysed with one-way ANOVA.

The blot shown in Figure 9 indicates that transfection was successful. Compared to the control samples (W, LP, and Neg), the silencing of TG2-L was pronounced, while the expression of TG2-S appeared to be maintained. The negative control (transfection with non-targeting siRNA) was included to validate the functional siRNA used. The bar graphs illustrate the expression levels of TG2-L (Figure 9b) and TG2-S (Figure 9c). TG2-L expression is consistent across the MCF control, lipofectamine-treated (LP), and negative control (Neg) groups, and in the siRNA MCF group, where TG2 was silenced, and a significant reduction in TG2-L expression was observed. In contrast, Figure 9c shows that TG2-S expression remained relatively stable across all groups, including the siRNA MCF group, which showed no significant reduction. This suggests that the siRNA employed here primarily affected the expression of TG2-L, while TG2-S levels were maintained, allowing us to highlight the potential role of TG2-L in regulating resistance to cisplatin.

**Figure 10:**
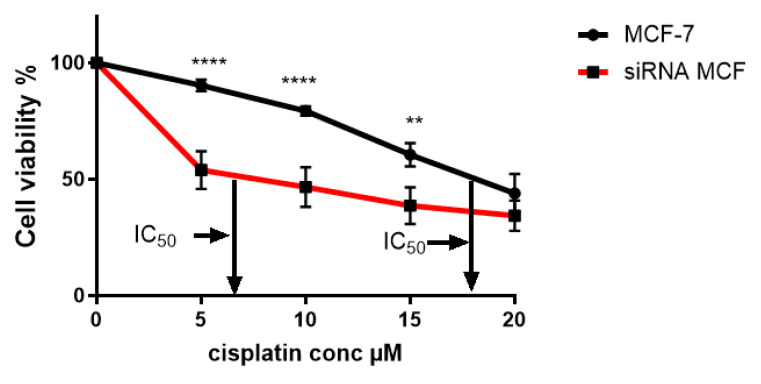
The cytotoxicity of cisplatin after transfection with anti-TG2 siRNA on wild-type MCF-7 cells. The cytotoxicity of cisplatin on transfected cells was determined using the CCK-8 assay, and results were computed from the mean of three independent experiments (±SEM). Statistical significance is denoted by **** (<0.0001) and ** (0.0011).

Following siRNA treatment of wild-type MCF-7 cells, the IC_50_ for cisplatin was determined to be 6 µM (Figure 10), which is a significant reduction from the value of 18 µM that was observed in wild-type MCF-7 cells not treated with siRNA targeting TG2. This suggests a relationship between TG2-L expression levels and the cells’ sensitivity to cisplatin.

**Figure 11:**
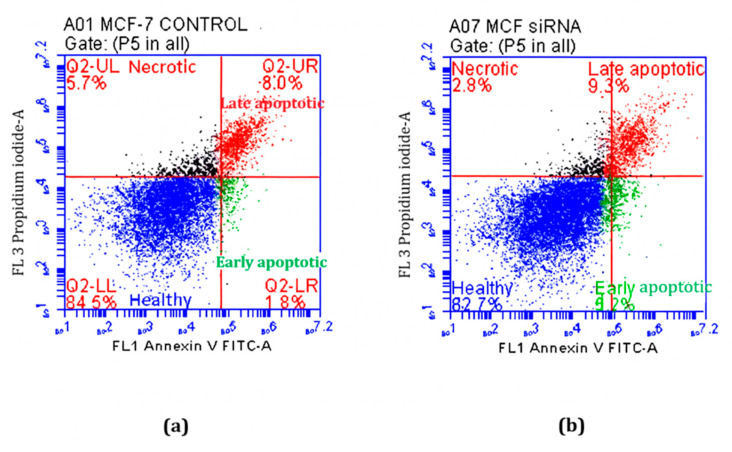
The flow cytometry analysis of wild-type MCF-7 cells after siRNA transfection. Representative cytogram of (**a**) control cells and (**b**) anti-TG2 siRNA-transfected cells. Quadrants indicate: viable cells (blue, Q2-LL), early apoptotic (green, Q2-LR), late apoptotic (red, Q2-UR), and necrotic cells (purple, Q2-UL). Most cells remained viable post-transfection.

After transfection, the cells were harvested and analysed using flow cytometry to assess their viability. Figure 11 indicates that the cells remained healthy post-transfection, confirming that transfection was not harmful to the cells.

**Figure 12:**
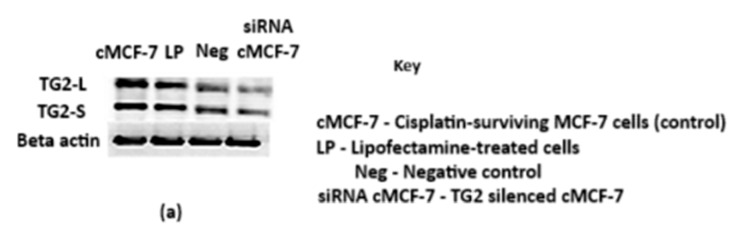

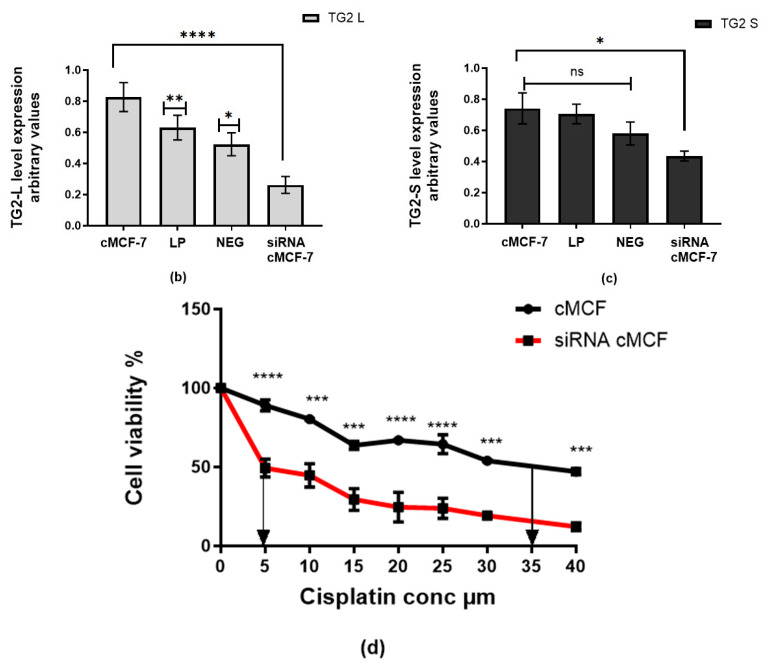
The effect of silencing on TG2 levels in cisplatin-surviving MCF-7 cells. (**a**) A representative Western blot analysis of TG2-L and TG2-S expression levels in cMCF-7 cells across different conditions. β-actin was used as a loading control. The histogram shows the quantification of TG2-L (**b**) and TG2-S (**c**) protein levels in cMCF-7 cells (untreated control), the lipofectamine-only control (LP), negative control siRNA (NEG), and TG2-specific siRNA-transfected cells (si-cMCF-7). Normalisation was performed with β-actin. Data represent the mean ± SEM (*n* = 3). One-way ANOVA revealed a significant difference in TG2-L expression across the groups (F(3,12) = 22.91, *p* < 0.0001). Dunnett’s post hoc analysis showed a substantial reduction in TG2-L levels in siRNA MCF-7 (**** *p* < 0.0001) compared to untreated cMCF-7 controls. For TG2-S, one-way ANOVA also indicated a significant difference (F(3,12) = 8.34, *p* = 0.0029), with siRNA cMCF-7 showing a statistically significant reduction compared to the control (*p* = 0.0417), whereas lipofectamine alone and the negative control siRNA showed non-significant differences (ns). These results confirm that TG2-specific siRNA effectively reduces both TG2 isoforms, with a more pronounced effect on TG2-L. (**d**) The cytotoxicity of cisplatin after transfection with anti-TG2 siRNA on cMCF-7 cells. The cytotoxicity of cisplatin on transfected cells was determined using the CCK-8 assay, and results were computed from the mean of three independent experiments (±SEM). Statistical significance is denoted by: ns = not significant, * *p* < 0.05, ** *p* < 0.01. *** (0.0005) and **** (<0.0001).

Cisplatin-surviving cells were also transfected with anti-TG2 siRNA, and the Western blot (in Figure 12a) and blot quantification (Figure 12b,c) present TG2-L and TG2-S expression levels under different conditions. The control (cMCF-7) was lipofectamine-treated (LP), and negative control (Neg) groups showed consistent TG2-L expression, indicating that transfection reagents and non-targeting siRNA do not significantly affect TG2-L levels.

In the siRNA-treated cMCF-7 group, where TG2 was specifically silenced, a marked reduction in TG2-L expression was observed (Figure 12b), confirming the effective knockdown of the long isoform. There was also a statistically significant reduction in the TG2-S isoform (Figure 12c), although to a more modest extent; the knockdown of TG2-L was more effective than the knockdown of TG2-S.

The effect of TG2-L isoform silencing on the IC_50_ of cisplatin that was observed in wild-type cells (Figure 10) was also seen—to a striking degree—in cisplatin-surviving cells (Figure 12). Interestingly, the IC_50_ of cisplatin in both wild-type and cisplatin-surviving cells was reduced to almost exactly the same value—around 5 µM—from IC_50_ values of 18 µM and 35 µM, respectively, when treated with anti-TG2 siRNA. This result supports the link between TG2-L upregulation and decreased cisplatin efficacy.

## 3. Discussion

Cisplatin has been extensively tested and has proven to be an effective drug, but it has some significant drawbacks for cancer patients. Whilst it is widely available and relatively affordable, there are challenges associated with its toxicity and its ability to cause the rapid induction of cancer cell resistance during chemotherapy. Exploring other aspects of patients’ treatment, such as their dietary regime, may, therefore, be necessary to maximise cisplatin’s anticancer potential.

The results of this study show that RA induces TG2 expression in MCF-7 breast cancer cells, which is consistent with previous studies on other cancer cells, including those derived from the liver, cervix, and prostate. The present study also reveals that the overexpression of one isoform, i.e., TG2-L, is more strongly linked to chemoresistance than the short TG2 form. However, it is important to consider that the ratio between TG2 isoforms may be a key factor in determining chemoresistance rather than the expression levels of a single isoform alone. Similar chemoresistance effects have been reported in other cancer cell line types, including hepatocarcinoma cells [15], ovarian cancer cells [29], and non-small-cell lung cancer cells [30], suggesting that the effect is not restricted to breast cancer or the MCF-7 cell line in particular.

Extensive research has explored the use of RA in preventing and treating various types of cancer [31,32,33,34,35,36], and studies have shown that using retinoids can clinically reduce disease recurrence and improve treatment response. For example, a meta-analysis involving 15,627 patients was conducted between 2000 and 2021 to determine the efficacy of retinoids in cancer chemoprevention and treatment. Retinoids significantly reduced disease recurrence and improved clinical response in acute promyelocytic leukaemia, renal cell carcinoma, hepatocellular carcinoma, lung cancer, Kaposi sarcoma, and the complete hydatidiform mole, but no significant positive impact was observed in cancers of the head and neck, AML, melanoma, breast, bladder, and cervix [37].

While phytochemicals are generally considered beneficial for preventing cancer in healthy individuals, they may have unexpected and unwanted side effects during chemotherapy [35,36,37,38]. RA provides such a case in point: it has demonstrated anti-tumour properties in some oestrogen receptor-positive breast cancers, but excessive levels may have opposing effects, potentially influencing cancer progression or stem cell behaviour. There has been particular controversy surrounding the use of RA in cancer treatment [39], as it has been shown to promote cell growth in some cases [40]. These effects are exerted through various binding proteins and nuclear receptors, including oestrogen receptor α (ERα) [41] and other related subtypes. The activation of ERα through the classical genomic pathway promotes cell cycle arrest, cell differentiation, and apoptosis [42]. In contrast, the activation of the non-classical pathway may modulate downstream gene expression by regulating other transcription factors such as NF-κB, as well as retinoic acid receptor α-forming heterodimers with ERα [43] and peroxisome proliferator-activated receptors (PPARs) [44]. These pathways are known to regulate cellular processes that are contrary to classical pathways. While the activation of RARs by RA often results in cell growth inhibition, it may promote cell survival and hyperplasia in specific tissues [40]. Interestingly, TG2 is also known to upregulate NF-κB in a mutually reinforcing positive feedback loop [43], lending further weight to the idea that cancer cells experiencing stimulation from both RA and TG2-L may be upregulated in survival and growth.

More broadly, it is now clear that TG2 plays a key role in chemoresistance in a variety of cancers, and it is therefore a promising therapeutic target. However, interventions need to ensure that the modulation of TG2 expression and/or enzyme activity does not carry risk in themselves. So far, clinical investigations with TG2 inhibitors have yielded positive outcomes. For example, AA9 has demonstrated the ability to reduce cancer cell migration, invasion, and survival, particularly in aggressive subtype models such as MDA-MB-231 [45]. Other research with the TG2 inhibitor ZED1227 in patients with coeliac disease demonstrated that the inhibitor was not harmful; ZED1227 effectively reduced gluten-induced mucosal damage without significant adverse effects [46].

While direct clinical studies focusing on TG2 silencing in patients are currently limited, preclinical research has provided valuable insights into the potential therapeutic benefits of targeting TG2 in various cancers. In vivo experiments have demonstrated that combining TG2 silencing with chemotherapy significantly inhibited tumour growth in mouse models, suggesting a promising therapeutic strategy [47]. Similarly, in acute myeloid leukaemia (AML), silencing TG2 in AML cells sensitised them to chemotherapy both in vitro and in vivo, leading to extended survival in leukaemia-bearing mice [48]. The differential effects of modulating TG2 short and long isoform expressions in vivo are yet to be investigated.

Interestingly, in the present study, the expression of TG2 isoforms did not directly mirror changes in enzymatic transaminating activity. There was no significant difference in TG2 activity between the RA-pre-treated wild-type MCF-7 cells and those without RA pre-treatment (Figure 8). Almost the same pattern was observed in the cisplatin-surviving cells, except notably at the 20 µM RA pre-treatment concentration, where the enzyme activity was double that of both the wild-type and the cisplatin-surviving cells without RA pre-treatment. The increase in TG2 activity in cisplatin-surviving MCF-7 cells at high RA concentrations (20 µM) suggests the potential role of TG2 enzyme activity—rather than expression per se—in mediating resistance mechanisms, unlike in wild-type MCF-7 cells, which exhibit minimal changes in TG2 activity across treatments. Given that TG2-L is associated with cell survival, its increased expression and activity in response to these high levels of RA could contribute to the reduced efficacy of cisplatin in inducing cell death; this is corroborated by our finding that the silencing of TG2-L expression significantly reduces the IC_50_ of cisplatin in both wild-type and cisplatin-surviving MCF-7 cells. This finding raises important considerations regarding the therapeutic use of high doses of RA, or diets rich in RA and its precursors, as such approaches may inadvertently support resistance pathways in certain cancer cells resembling MCF-7. We do not advocate RA restriction as a therapeutic strategy but propose that further investigation into the relationship between exogenous RA exposure and TG2 expression may inform more nuanced dietary or pharmacological guidance in the future.

Further investigation is needed to determine whether this increase in TG2 activity directly enhances survival signalling or alters drug response mechanisms, which could have implications for treatment strategies combining RA with chemotherapy.

### Limitations

In addressing the potential limitations of this study, we acknowledge that MCF-7 is a hormone receptor-positive breast cancer cell line and that cisplatin is more commonly associated with triple-negative breast cancer treatment. However, as outlined in the introduction, emerging evidence supports the relevance of cisplatin as a treatment for hormone-positive disease in some cases, and this study aims to contribute to this specific and somewhat under-researched area. Our choice of MCF-7 was informed by its well-characterised phenotype and widespread use in mechanistic studies [49], including those exploring retinoic acid (RA) signalling and TG2 expression. These findings support the use of the MCF-7 ER⁺ model in studying cisplatin response and resistance mechanisms that are contextually relevant to hormone-positive disease.

While this study was limited to a single cell line, the in-house development of a cisplatin-surviving MCF-7 sub-line (cMCF-7) strengthened the reliability of our findings; this sub-line model demonstrates decreased cisplatin sensitivity compared to the parental line, as evidenced by the viability assays and migration patterns shown in Figure 1, Figure 2 and Figure 3. However, acknowledging the limitations of using a single parental cell line, we recommend that future work include validation with additional hormone-positive cell lines and extension to triple-negative models and patient-derived organoids.

While RA treatment consistently upregulated TG2-L expression, the modulation of TG2-S was variable and did not reach statistical significance across all replicates. This variability suggests that different mechanisms, or thresholds of RA responsiveness, may regulate TG2-S. Further studies using isoform-specific quantification techniques and gene silencing approaches will be necessary to define the distinct roles and regulation of TG2-L and TG2-S in chemotherapy response.

Indeed, the differential expression and localisation of TG2 isoforms, particularly TG2-L and TG2-S, appear to influence cisplatin response; however, the precise regulatory mechanisms require further investigation. Expanding this research to additional breast cancer subtypes and validating the clinical utility of the TG2 isoform measurement may improve strategies for overcoming chemotherapy resistance.

We also acknowledge that pharmacological concentrations of RA used in vitro may exceed physiological levels; in plasma, atRA levels typically have a range of 50–3200 pg/mL (0.167–10.65 nM)) [50], though dietetic studies have shown that under normal conditions, breast tissue can accumulate the RA precursor retinol to concentrations as high as 1–2 µM, and that dietary supplementation can transiently elevate retinol in breast milk to approximately 8 µM within 24 h, before gradually returning to baseline [51]. Concentrations similar to those used in this paper have been used to investigate mechanistic pathways [52,53,54], but care should be taken with the extrapolation of such mechanistic studies to patient scenarios.

RA is a well-characterised transcriptional regulator of TG2, allowing us to explore endogenous regulatory mechanisms within a broader physiological signalling context. However, while RA was employed in this study as a tool to modulate endogenous TG2 expression, it is important to acknowledge that RA regulates a broad range of gene targets through RAR/RXR-mediated transcriptional control, making it a pleiotropic agent with limited specificity [55,56,57]. Although RA is widely used experimentally for mechanistic studies of TG2 function, this raises concerns regarding RA’s clinical utility as a therapeutic modulator of TG2 alone.

In contrast, TG2 has been directly implicated in drug resistance, cell death, and fibrosis, and its activity varies depending on its localisation and conformation, making it a promising and precise therapeutic target [4]. Our finding that the siRNA-mediated silencing of TG2 reduces cisplatin IC_50_ in both wild-type and cisplatin-surviving MCF-7 cells supports the therapeutic potential of TG2-specific modulation. Therefore, future research should prioritise direct TG2 targeting through isoform-specific inhibitors or gene silencing over indirect RA modulation to isolate and validate TG2’s role in chemoresistance more precisely. While RA was used here to modulate TG2 indirectly, future studies could explore the effects of TG2-specific inhibitors or gene silencing to confirm target specificity and avoid potentially non-physiological doses of RA. In parallel, we suggest that future studies evaluate TG2 regulation and RA-induced chemoresistance at physiologically relevant concentrations of RA and use in vivo models.

## 4. Materials and Methods

### 4.1. Pharmacologic Agents

All reagents were obtained from Sigma Aldrich (Merck Life Science UK Limited, Gillingham, Dorset, UK) unless otherwise stated.

A fresh 2 mM working stock solution of cisplatin (cis-diammineplatinum (II) dichloride) was prepared in the dark by dissolving the powdered product (CAS 15663-27-1) in double-distilled water, followed by filter sterilisation using a 0.2 µm syringe filter for each experiment [58]. Likewise, a 10 mM RA stock solution was prepared under subdued lighting in a glove bag by dissolving 3 mg/mL in absolute ethanol, as RA is sensitive to UV, air and oxidising agents. Aliquots were then stored at −20 °C.

### 4.2. Cell Culture

The human breast cancer cell line, MCF-7, was obtained from the American Type Culture Collection (ATCC; Manassas, VA, USA) and cultured in Dulbecco’s Modified Eagle Medium (DMEM) from Sigma Aldrich (Merck Life Science UK Limited, Gillingham, Dorset, UK). The medium was supplemented with 10% foetal bovine serum (FBS) and 1% penicillin-streptomycin (Invitrogen, Paisley, UK). Cells were grown and maintained in a humidified atmosphere of 5% CO_2_ at 37 °C.

### 4.3. Development of a Cisplatin-Surviving MCF-7 Sub-Line

The IC_50_ of the original parental (MCF-7) cell line was determined by the exposure to cisplatin for 24 h with concentrations informed by initial dose–response studies of cisplatin (0–50 µM). Cisplatin-surviving (cMCF-7) variants were exposed at the IC_50_ of wild-type cells for 24 h; the growth medium was replaced with a drug-free medium until the cells attained 80% confluence. This process of the drug challenge/selection was repeated twice, and surviving cells were subsequently maintained in a drug-free medium with appropriate supplements until confluent.

The IC_50_ of the wild-type cells was compared with that of the cisplatin-surviving cMCF-7 cells, and the disparity between the cell lines indicated the level of reduced sensitivity to cisplatin.

### 4.4. Cell Viability Assay/IC_50_ Toxicity Assay

Cell viability and proliferation were determined through sensitive colorimetric assays (CCK-8 and MTT). Cells were seeded in triplicate in 96-well plates at a concentration of 10^5^ per well and allowed to adhere. They were incubated overnight at 37 °C and 5% CO_2_ in a humidified atmosphere. The response to chemotherapeutic agents was found by determining the minimum inhibitory concentration (IC_50_)—the drug concentration required to inhibit 50% of the cells from growing [59]. The IC_50_ was found using a dose–response curve.

### 4.5. Wound-Healing “Scratch” Assay

A wound-healing assay [60] was performed by growing approximately 5 × 10^5^ cells in a 6-well plate for 24 h. The medium was replaced with a fresh medium, and cells were allowed to grow until they became confluent. A “scratch” or “wound” was made on the cell monolayer using a 10 µL pipette tip, and images of the cells were captured at different time points as the “scratch” healed for 24 h; the width of the remaining “scratch” was measured at each time point. Images were captured at 0, 2, 4, 8, 12, and 24 h with the ZOE™ Fluorescent Cell Imager (Bio-Rad, Hercules, CA, USA). The rate of cellular migration was computed by measuring the “wound” width in micrometres (µm) with the ImageJ software version 2.14.0 (Fiji/ImageJ, National Institutes of Health, Bethesda, MD, USA).

### 4.6. Treatment with Retinoic Acid and/or Cisplatin

To assess the effect of increased TG2 levels on cell sensitivity to cisplatin, wild-type cells pre-treated with RA were exposed to cisplatin (IC_50_ concentration). Briefly, cells were seeded in a 96-well plate, treated with 0–20 µL of RA for 72 h, replaced with a medium containing/not containing cisplatin, and incubated for 24 h. After the incubation period, the viability of cells was determined using the MTT assay and analysed by flow cytometry to determine the phases of apoptosis present.

### 4.7. Tissue Transglutaminase Enzyme Activity

The specific tissue transglutaminase colorimetric microassay (NBP1-37008 by Novus Biological, Cambridge, UK) was used to determine the enzyme activity using whole protein lysate. The optical density was measured at 450 nm using the automated microplate reader ELx 800 (BioTek, Cheshire, UK). The assay was performed according to the manufacturer’s instructions.

### 4.8. TG2 Inhibition by siRNA

TG2 inhibition with specific anti-TG2 siRNA was performed using pre-designed siRNA forward (5′GGCCCGUUUUCCACUAAGATT3′) and reverse (3′UCUUAGUGGAAAACGGGCCTT5′) sequences and transfection with the lipofectamine 2000 transfection reagent. Reagents were obtained from Life Technologies, Thermo Fisher Scientific, Paisley, UK. Cells were seeded into 96-well plates (1 × 10^4^ per well) for viability assays and 6-well plates (1 × 10^5^ per well) for protein extraction and were allowed to adhere to the plates. The negative control was scrambled (non-targeting) siRNA. After 24 h, according to the manufacturer’s guidelines, the medium was replaced by Opti-MEM™ medium, with reduced FBS because the serum can affect transfection results. The siRNA was added to 100 µL of the medium for a final concentration of 30 pM. Cells were incubated for 48 h and used for further analysis.

### 4.9. Apoptosis Analysis

We used the Annexin V-FITC assay (BD Biosciences, San Diego, CA, USA) and a FACS Calibur flow cytometer (BD Biosciences, Erembodegem, Belgium) for all flow cytometry analysis. Wild-type and cisplatin-surviving cells were grown to about 70% confluence, trypsinised, seeded at 1 × 10^5^ cells in each well of 6-well plates, and allowed to adhere to the plates for 24 h. Cells were treated with either cisplatin (24 h) or RA (72 h). After incubation, cells were collected by trypsinisation, pelleted by centrifugation at 1500 rpm for 5 min, washed, and prepared for analysis. The cells were then analysed for apoptosis following the manufacturer’s directives by adding 5 µL of Annexin V-FITC and 5 µL propidium iodide (PI) to the cells in the binding buffer before incubating in the dark for 15 min. After incubation, 300 µL of a binding buffer was added, and the samples were analysed. Gating (the selection of an area on the scatter plot with a specific population of cells) locates viable single-cell events using the forward scatter (FSC) and side scatter (SSC) on the scatter plot. The gate excluded events with low FSC, representing cellular debris, and high SSC, which are doublets, from the analysis.

### 4.10. Western Blot Analysis

Total protein was extracted from wild-type and cisplatin-surviving cells (around 1 × 10^6^ cells per sample) using an ice-cold radioimmunoprecipitation assay buffer (RIPA buffer) supplemented with protease inhibitors (Sigma Aldrich, UK). The electrophoresis of whole protein lysate (50 µg) was carried out using 10–12% NuPAGE Bis-Tris mini gels with the MES Running Buffer (Invitrogen) at 150 V and 100 mA. After separation, proteins were transferred onto nitrocellulose membranes at 15 V and 60 mA for 1 h, and then the membranes were blocked with 2% *w*/*v* bovine serum albumin solution for 1 h. The membranes were washed thrice with TBS-Tween 20 (TBST) before probing with a TG2 primary antibody (mouse anti-TGM2 CUB 7402) at a dilution of 1:3000 at 4 °C overnight. Membranes were then washed thrice with TBST before further incubation with secondary antibodies (goat anti-mouse-HRP conjugate (ab07023)) at a dilution of 1:4000 for 1 h at room temperature. The anti-β-actin antibody (AC-15) (ab6276) was used to control for loading at a 1:3000 dilution. Developing the membranes involved washing thrice with TBST before developing horseradish peroxidase (HRP). Membranes were developed with the Clarity Western ECL Substrate (Bio-Rad Watford, Hertfordshire, UK). Membranes were covered with equal volumes of Clarity Western peroxide reagent and Clarity Western luminol/an enhancer reagent and left at room temperature for 3 min. Excess fluid was drained, and membranes were air-dried for digital imaging with the Syngene GBOX CHEMI XRQ (Syngene, Synoptics Ltd., Cambridge, UK).

### 4.11. Statistical Analysis

Data generated from the experiments were analysed with GraphPad Prism software version 7.0 and expressed as the mean ± standard error of the mean (SEM). Student’s *t*-test and 2-way ANOVA at a *p* < 0.05 determined significant differences between the control and treated groups. The results were analysed from three independent experiments.

## 5. Conclusions

In this study, MCF-7 cells were treated with 10–20 µM of RA to explore the mechanistic effects. While these in vitro concentrations are much higher than typical plasma levels, the findings may have implications for patients supplementing their diet with commercially available vitamin preparations and possibly for those using topical applications of retinoic acid in cosmetics, for which the long-term effects over months or even years appear under-investigated. While our experimental doses exceeded these levels, they fell within the range commonly used in mechanistic in vitro studies [61] to ensure sufficient modulation of gene expression pathways, including the induction of TG2. Understanding the relationship between retinoid intake and chemoresistance in a clinical setting could help refine dietary recommendations for individuals undergoing chemotherapy, ensuring that retinoid levels remain within a range that does not compromise treatment efficacy.

Further, clinical trials assessing the impact of TG2 suppression on patient outcomes provided valuable insights into its potential as a therapeutic target. Indeed, the significance of differential isoform expressions and the enzyme activity of TG2 in cancer is by no means the only aspect to consider when investigating the roles of TG2 in cancer pathology. For example, our group recently investigated the extracellular and intracellular localisation of TG2 expression in invasive breast cancer in a cohort of 2169 cases to determine the prognostic role of TG2. Intracellular TG2 expression in tumour cells was associated with better patient survival rates, especially in hormone-receptor-negative cases, indicating a potential protective role. Extracellular TG2 expression was associated with poorer prognosis in hormone-receptor-positive cases, suggesting its involvement in adverse tumour microenvironment interactions [62]. Another study from our group showed that the localisation of the TG2 isoforms regulates their functions, with TG2-S playing a key role in cell death upon their release from membrane-bound pools; indeed, the measurement and distribution of TG2 isoforms may be biomarkers for optimising cisplatin therapy [63].

Further studies are needed, both in this breast cancer model and other types of in vitro and in vivo models, to explore holistically how the interplay between TG2-L and TG2-S influences drug response and disease progression.

## Data Availability

The original contributions presented in this study are included in the article/Supplementary Materials. Further inquiries, including the raw data supporting the conclusions of this article, can be made to the corresponding author.

## Supplementary Materials

The following supporting information can be downloaded at https://www.mdpi.com/article/10.3390/ijms26168101/s1.

## Abbreviations

The following abbreviations are used in this manuscript:

RA	Retinoic Acid
RARs	Retinoic Acid Receptors
RXRs	Retinoid X Receptors
RAREs	Retinoic Acid Response Elements
atRA	All-Trans Retinoic Acid
TG2	Tissue Transglutaminase 2
TG2-L	Tissue Transglutaminase 2, Long Isoform
TG2-S	Tissue Transglutaminase 2, Short Isoform
Ca^2+^	Calcium Ions
GTP	Guanosine Triphosphate
MCF-7	Michigan Cancer Foundation-7 (a breast cancer cell line)
ERα	Oestrogen Receptor Alpha
NF-κB	Nuclear Factor Kappa-Light-Chain-Enhancer of Activated B Cells
PPARs	Peroxisome Proliferator-Activated Receptors
AML	Acute Myeloid Leukaemia
CCK-6	Cell Counting Kit-6
IC_50_	Half Maximal Inhibitory Concentration
cMCF-7	Cisplatin-surviving MCF-7 Cells
AA9	A TG2 Inhibitor
DMEM	Dulbecco’s Modified Eagle Medium
FBS	Foetal Bovine Serum
ATCC	American Type Culture Collection
CO_2_	Carbon Dioxide
MTT	Methylthiazolyldiphenyl-tetrazolium Bromide
ZOE™	Fluorescent Cell Imager (Brand)
FITC	Fluorescein Isothiocyanate
PI	Propidium Iodide
FACS	Fluorescence-Activated Cell Sorting
FSC	Forward Scatter
SSC	Side Scatter
RIPA	Radioimmunoprecipitation Assay Buffer
TBST	TBS-Tween 20
HRP	Horseradish Peroxidase
ECL	Enhanced Chemiluminescence
