# Supplementary material for: Retinoic Acid-Induced Transglutaminase 2 Expression Reduces Sensitivity to Cisplatin in the Hormone-Positive MCF-7 Breast Cancer Cell Model
Source: Int J Mol Sci. 2025 Aug 21;26(16):8101. doi: 10.3390/ijms26168101 (PMC12387040; doi:10.3390/ijms26168101)
Supplement: Supplementary file 1 [file ijms-26-08101-s001.zip › ijms-3515765-supplementary.pdf]

Supplementary Materials

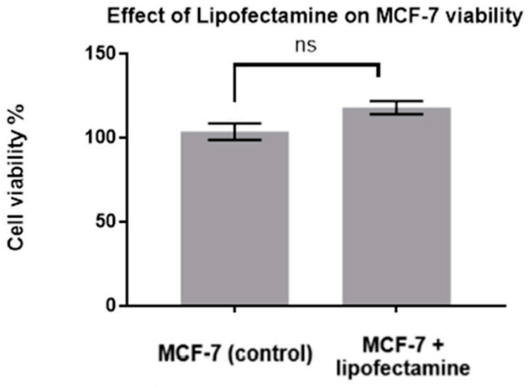

Supplementary Figure S1. Viability of MCF-7 cells after lipofectamine treatment

The CCK-8 assay determined the cell viability of lipofectamine-treated cells and non-treated control cells. Results were computed from at least three independent experiments (±SEM), and the difference was not statistically significant when analysed with the Student T-test.

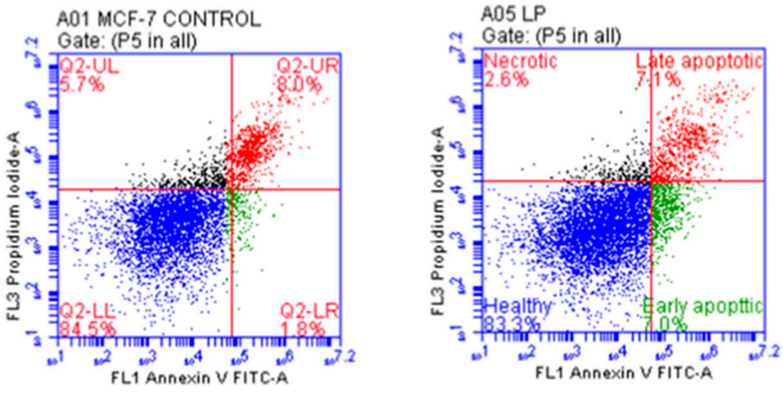

(a) (b)

Supplementary Figure S2. Cytometric analysis of lipofectamine-treated cells

Representative cytogram of cells treated/not treated with lipofectamine. SSC-A/FSCA was employed to collect single cells, with gating to exclude cell debris and doublets. Representative cytogram of (a): MCF-7 control cells, and (b): MCF-7 lipofectamine-treated cells.
